# HIV-1 V3 envelope deep sequencing for clinical plasma specimens failing in phenotypic tropism assays

**DOI:** 10.1186/1742-6405-7-4

**Published:** 2010-02-15

**Authors:** Ina Vandenbroucke, Herwig Van Marck, Wendy Mostmans, Veerle Van Eygen, Evelien Rondelez, Kim Thys, Kurt Van Baelen, Katrien Fransen, Dolores Vaira, Kabamba Kabeya, Stephane De Wit, Eric Florence, Michel Moutschen, Linos Vandekerckhove, Chris Verhofstede, Lieven J Stuyver

**Affiliations:** 1 Tibotec-Virco Virology BVBA, Mechelen, Belgium; 2 Department of Microbiology, Institute of Tropical Medicine, Antwerp, Belgium; 3 Aids Reference Laboratory and Aids Reference Center, University of Liège, CHU Sart Tilman, Liège, Belgium; 4 Department of Infectious Diseases, St Pierre University Hospital, Brussels, Belgium; 5 Department of Clinical Sciences, Institute of Tropical Medicine, Antwerp, Belgium; 6 Aids Reference Laboratory, Ghent University Hospital, Ghent, Belgium; 7 General Internal Medicine, Infectious Diseases and Psychosomatic Disorders department, Ghent University Hospital, Ghent, Belgium

## Abstract

**Background:**

HIV-1 infected patients for whom standard gp160 phenotypic tropism testing failed are currently excluded from co-receptor antagonist treatment. To provide patients with maximal treatment options, massively parallel sequencing of the envelope V3 domain, in combination with tropism prediction tools, was evaluated as an alternative tropism determination strategy. Plasma samples from twelve HIV-1 infected individuals with failing phenotyping results were available. The samples were submitted to massive parallel sequencing and to confirmatory recombinant phenotyping using a fraction of the gp120 domain.

**Results:**

A cut-off for sequence reads interpretation of 5 to10 times the sequencing error rate (0.2%) was implemented. On average, each sample contained 7 different V3 haplotypes. V3 haplotypes were submitted to tropism prediction algorithms, and 4/14 samples returned with presence of a dual/mixed (D/M) tropic virus, respectively at 3%, 10%, 11%, and 95% of the viral quasispecies. V3 tropism prediction was confirmed by gp120 phenotyping, except for two out of 4 D/M predicted viruses (with 3 and 95%) which were phenotypically R5-tropic. In the first case, the result was discordant due to the limit of detection for the phenotyping technology, while in the latter case the prediction algorithms were not computing the viral tropism correctly.

**Conclusions:**

Although only demonstrated on a limited set of samples, the potential of the combined use of "deep sequencing + prediction algorithms" in cases where routine gp160 phenotype testing cannot be employed was illustrated. While good concordance was observed between gp120 phenotyping and prediction of R5-tropic virus, the results suggest that accurate prediction of X4-tropic virus would require further algorithm development.

## Background

The chemokine receptors CCR5 and CXCR4 are the main co-receptors for entry of HIV-1 into target cells [1,2]. Maraviroc (Selzentry/Celsentri, Pfizer, NY) is a chemokine co-receptor antagonist, designed to prevent HIV-1 infection of CD4^+ ^T-cells by blocking the CCR5 co-receptor. Since the drug is only effective in individuals exclusively harboring CCR5-tropic (R5) virus, viral tropism has to be determined before the initiation of maraviroc treatment. Currently, the only clinically validated tropism test is the Trofile assay (Monogram Biosciences, CA). It has recently been replaced by the Enhanced Sensitivity Trofile Assay (ESTA), which detects minority CXCR4-using (X4) viruses with higher sensitivity in clinical specimens [3]. However, the use of this type of phenotypic assays has several limitations: (i) the need to perform these assays in a centralized lab; (ii) the limited amplification success rate of gp120 (Virco tropism assay) or gp160 (Trofile assay) envelope gene, and (iii) the relatively long turn-around times, high cost, and requirement for large fresh specimen. There is an ongoing search for alternatives [4-6], most commonly relying on the amplification of the V3 domain of gp120, which is the major determinant for viral tropism [7,8]. Prediction of co-receptor usage based on V3 sequences using bioinformatics tools could be a good alternative for phenotypic tropism testing in routine clinical practice [9-11]. However, due to the lack of sensitivity of standard sequencing methods, the use of predictions based on population sequencing may be misleading. Massively parallel sequencing technologies allow sensitive, quantitative, and clonal analysis of sequence variability. When combined with genotypic prediction tools, they could become a sensitive alternative to phenotypic assays.

## Results

### Assay performance

**Figure 1:**
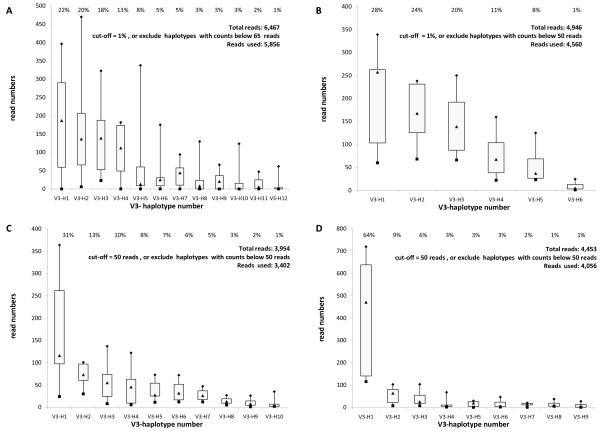
**Boxplots illustrating quasispecies variability for the V3-loop of 4 independent clinical isolates**. X-axis: haplotypes present in a clinical isolates, with a cut-off of 50 reads or 1%. Y-axis: number of reads from the GS-FLX amplicon sequencing reaction. Boxplots represent sequence read numbers of 7 individual experiments. Boxplots symbols are as follows: black squares = min; bottom of the box = interquartile range 1; black triangles = median; top of the box = interquartile range 3; and black diamonds = max. Example of an haplotype read distribution of the 7 parallel experiments for V3-H2 of sample A: 6, 11, 120, 137, 182, 232, and 470; resulting in the following numbers for the boxplot: min: 6, q1: 65,5, median: 137, q3: 206,9, and max: 470; total number of reads: 1157.

The assay principle is based on parallel reverse transcription and amplification of seven viral RNA aliquots, followed by pooling of the obtained amplicons which are subsequently sequenced. In order to illustrate that the approach of pooling replicates could reduce the founder effect of the RT-PCR procedure, we sequenced four samples with high viral load (>4 log_10_) from unrelated clinical cases without amplicon pooling. Each independent RT-PCR reaction was sequenced separately and analyzed for quasispecies variability (Figure 1). To exclude variability due to technical error and not to viral genetic variability, we selected a cut-off of 1% (total read number > 5,000) or 50 reads (less than 5,000 reads in total), thereby discarding all variability at lower read frequencies. Using these stringent criteria, it was assumed that the observed haplotypes represent true viral variants, and not sequencing errors. Analysis of the data lead to several conclusions: i) the representation of specific haplotypes varies considerably among the seven replicates (Figure 1: see ranges of the boxes; e.g., range for sample A-V3-H2 from 6 to 407 reads - details in Figure 1 legend; range for sample D-V3-H1 from 141 to 719 reads; ii) on average, the 1% or 50-read limit retains the top 90 ± 3% of reads; iii) expressing an haplotype percentage is most probably more balanced if derived from pooling approach; and iii) for a given sequence the same tropism prediction was retrieved independent from PCR conditions, but the quantity was found to be variable from one experiment to the other.

In conclusion, the range of haplotypes reads per samples varied considerably dependent on the RT-PCR experiment, and therefore the results demonstrate that pooling of multiple parallel independent amplification reactions reduces intra-assay variability. The average result of seven pooled reactions provides a more reliable quantification of each of the quasispecies. Therefore, this 7× repeat protocol was used as standard protocol.

### Tropism testing on clinical samples using deep sequencing

A total of 14 clinical samples from 12 different HAART failing individuals were available. Despite the clinical need for alternative drug regimens, maraviroc was not a treatment option because routine phenotypic tropism testing failed, thereby excluding these patients from co-receptor antagonist based regimens.

HIV-1 V3 GS-FLX sequencing of the clinical samples yielded an average of 12,698 ± 3,604 reads (range 3,370 to 19,626) per sample (Additional file 1: Overview Results). Since the viral load ranged from 3.2 to 5.81 log_10_, and the experiments started with 256 μl of plasma, a theoretical maximum of 396 up to 161,414 individual viral RNA copies was included in the reactions. In the current experimental set-up, with an average of 12,698 ± 3,604 reads, an oversampling ratio of ~1 would be obtained in samples with an original viral load of 4.7 log_10_. (or 50,000 cp/ml = 12,500 copies in 256 μl). Taking these calculations into account, the oversampling sizes in our experiments were ranging from 0.1 to 31.5. These are obvious consequences of the sample original viral load levels (Additional file 1: Overview Results).

The number of independent V3-loop haplotypes detected in these isolates depended on the sequence read number cut-off used (Additional file 1: Overview Results). If the cut-off of 50 reads was applied, a 7 ± 3 haplotypes were detected on average, while a 1% read cut-off reduced the average to 5 ± 3 haplotypes. Increasing the cut off can have a reducing effect on the number of X4 haplotypes (sample 11 and 12, Additional file 1: Overview Results). The number of haplotypes reaching 10% was on average 2, further reduced to 1 (exceptionally 2) haplotype above 25%.

**Figure 2:**
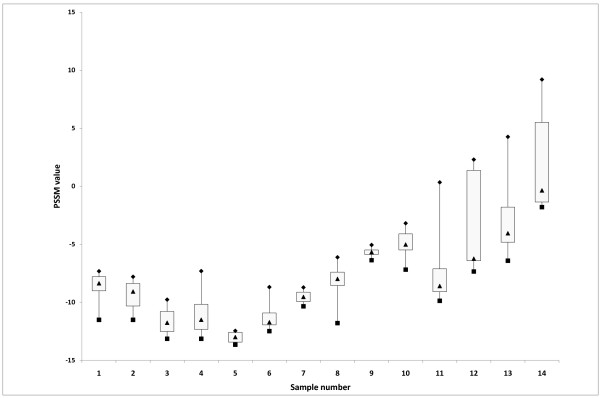
**Overview of the distribution of PSSM scores of the V3 haplotypes on 14 clinical isolates failing the gp160 phenotyping assay**. Boxplot symbols are as in Figure 1. Data (min, q1, median, q3, max) for this figure are given in Additional file 1: Overview Results.

Haplotype sequences from all patients were submitted to PSSM tropism prediction tool (Additional file 1: Overview Results, Figure 2). In samples 1 - 10 only R5-tropic haplotypes were observed. X4-tropic haplotypes were detected - with increasing prevalence - in samples 11 to 14. While in sample 11 and 12 the X4-tropic viruses were divided over two haplotypes each individually accounting for less than 10% of the population, this was not the case for sample 13 (one haplotype reached 10%), and also not for sample 14 for which all haplotypes (minor and major) were predicted to be X4-tropic by PSSM.

All haplotypes were also analyzed by the G2P prediction tool. Identical prediction outcomes as for PSSM were obtained for all, except for three X4-PSSM predicted minority haplotypes in sample 14 that were predicted to be R5-tropic in G2P, accounting in total for 4% of the population.

### Tropism testing on clinical samples using VTA

In order to verify the genotypic tropism predictions, a population tropism phenotyping test was performed on each sample, using a smaller fragment (NH_2_-V4 region) than the gp160 domain [12]. VTA was able to detect the presence of X4 virus in 2/4 samples with predicted X4 virus (D/M in Additional file 1: Overview Results). The 2 discordant samples (sample 11 and 14; Additional file 1: Overview Results) belonged to subtype C. Further analysis of the X4-predicted data from the PSSM algorithm showed percentile values (= value between 0 and 1, and indicating whether the sample resembles similar sequences in the training data-set) of more than 0.95, indicating that they did not resemble an algorithm-known V3 sequence, and therefore the limited reliability of the prediction might have been the explanation of the discordant result.

## Discussion

Samples might fail phenotypic tropism testing due to difficulties in amplification of the large envelope gene (sample degradation or low viremia), fitness problems or low infectivity of the recombinant virus. Our results demonstrate the potential use of massively parallel sequencing in cases where routine phenotype testing could not be employed. While the Trofile phenotyping assay is currently the only diagnostic tool for enrollment of patients in maraviroc-containing drug regimens, alternative 'genotypic' approaches should be considered and validated in clinical settings. It needs to be stated that other phenotyping approaches (e.g. shorter fragments like gp120) might be able to provide results where the Trofile assay fails, but these phenotyping technologies are not the most attractive options for routine clinical use because, in principle, they suffer from the same limitations (cost, turn-around time, centralized lab services). On the other hand, second generation sequencing technologies are opening new diagnostic avenues, but there are still some unresolved challenges associated to i) the methods (sample shipment, extraction, amplification and pooling, sequence alignments and interpretations), ii) the availability of the final product in a reproducible and standardized way, and iii) the cost per sample.

Clinical isolates can harbour multiple or just one major haplotype. In Figure 1, sample A and B show 4 haplotypes, each representing over 10% of the reads, while other clinical samples have only one major haplotype (e.g., sample D-V3-H1 represents 64% of all reads). The same observation was also illustrated in Additional file 1: Overview Results, where a range of 1 to 4 major haplotypes can be observed (cut-off dependent). Less sensitive sequencing technologies (Sanger approaches) are in general able to detect the major haplotypes, but not the minor ones, nor the linkage with other mutations. In this study, 4 samples with predicted X4-tropic haplotypes were found, an observation that would otherwise remain undetected. Using massively parallel sequencing technologies, the minor variants as well as haplotypes are detected and their relevance to the clinical aspects can be studied.

Using a standardized protocol in combination with a variable viral load between the different samples results unavoidably in under- or oversampling of the viral quasispecies pool. Despite this range in sample viral load, the number of haplotypes is rather constant. For example: sample 12 has an oversampling size of 0.1, while the amount of haplotypes with more than 50 reads was limited to 5, while sample 1 shows a high oversampling rate (31.5) and 9 haplotypes were present. The current analysis was based on V3 amino acid sequences, and not on nucleotide variability in gp120. Phylogenetic analysis of the gp120 nucleic acid variability could be used to evaluate the quantity of resampling (identical nucleotide sequences are most likely derived from the same founder virus). The present study, however, suggests that, at the amino acid level, there is a limited quasispecies variability for V3 and only a few haplotypes are relevant at a certain time in the life cycle of the virus.

Viruses from 4/12 patients who were HAART failing individuals were predicted as X4 tropic by V3 genotype prediction, of which 2 were confirmed by VTA. Given the treatment-experience and subsequent treatment failure, the number of patients harbouring CXCR4-using viruses in this study was relatively low. In addition, 2/3 subtype C viruses in this study were predicted as X4 virus, which is also different from previous reports where less X4 tropic virus was found in clade C. Based on these observations, it is likely that the set of clinical isolates is too limited to make strong conclusions on X4 prevalence in treatment failures or in clade C virus.

The discordant result between the phenotypic assay and the prediction tool observed for sample 11 and 14 illustrates the limitations of the approach. The clinical decision on the use of co-receptor antagonists is mainly driven on detecting the presence of X4-tropic virus, therefore these discordances are hampering the usefulness of the prediction tools. With future availability of correlative tropism genotype-phenotype databases enriched with X4-tropic virus, it is anticipated that these prediction technologies will become more accurate. For the other 12 samples in this study, the prediction algorithms were in line with the gp120 phenotype.

## Conclusions

It was shown that that massively parallel sequencing technologies allow a sensitive and quantitative analysis of V3 loop variability, and might represent an alternative for phenotypic tropism assays when combined with accurate prediction tools. However, since the application was illustrated on only a small set of samples, the clinical utility of deep sequencing-V3 prediction described in this manuscript requires further evaluation before using this protocol for patient treatment decisions. If confirmed, this will be beneficial for patients for whom phenotypic tropism test results could not be obtained and who were therefore unable to consider CCR5 antagonists as part of their antiretroviral drug regimen.

## Methods

### Patients samples

In the context of a larger study evaluating new diagnostic procedures for tropism determination (Vandekerckhove L. et al., in preparation), a set of clinical specimens from twelve HIV-1 infected individuals failing HAART therapy and also failing in the Trofile assay became available for massively parallel sequencing (Additional file 1: Overview Results). Two follow-up samples were available from two of the patients (samples 1 and 2 are related to the same patient, and 3 and 4 are related to another one), collected within a three-month interval in absence of changes in treatment regimen. The samples belonged to seven different viral subtypes, determined in the protease-reverse transcriptase region (B (n = 4), C (n = 3), G (n = 3), AE (n = 1), A1 (n = 1), A/AG (n = 1) and 19_cpx (n = 1)). Three viral load (VL) groups (3 < VL <4 log_10 _(n = 5), 4 < VL < 5 log_10 _(n = 8) and VL > 5 log_10 _(n = 1)) were represented in the cohort (Additional file 1: Overview Results).

For assay validation purposes, four unrelated plasma samples with viral load > 4 log_10 _were obtained from the Virco R&D sample repository.

### V3 loop massively parallel sequencing

Viral RNA was extracted from 256 μl of plasma (BioRobot MDx, Qiagen, Hilden, Germany), reverse transcribed to cDNA, and the V3 loop was amplified in a nested PCR using barcoded primers (HXB2 positions: forward primer 6986-7012, reverse primer 7520-7540). Addition of barcode sequences to the primers allowed the simultaneous processing of amplicons originating from multiple individuals, enlarging the number of reads obtained per sequencing experiment [13]. To maximize the number of input templates and to minimize variation due to PCR drift, the RNA derived from 256 μl of plasma was divided in 7 aliquots, and 7 parallel RT-PCR reactions were performed [14,15]. Barcoded amplicons were pooled equimolarly and sequenced on the GS-FLX instrument according to the manufacturer's amplicon sequencing protocol (454 Life Sciences, Branford, CT, USA).

### Tropism prediction

The V3 loop sequences were aligned using a Hidden Markov Model (HMM) and translated to amino acids for tropism prediction using two different algorithms. Viral tropism was predicted based on the V3 loop sequence using the PSSM algorithm http://indra.mullins.microbiol.washington.edu/webpssm/ and the geno2pheno prediction algorithm http://coreceptor.bioinf.mpi-inf.mpg.de/index.php[16] with a false positive rate of 5%.

### Sequence error characterization

The frequency and distribution of errors introduced during V3 amplification and GS-FLX sequencing was assessed by analyzing data from amplicon sequencing of 2 plasmids in duplicate. All changes relative to the published sequence detected were considered to be technical artefacts. An amplicon (in duplicate) covering the V3-region of HIV was created from plasmid pHXB2 [GenBank:K03455] and pYK-JRSCF [GenBank:AY426126]. V3 amplicons were sequenced using the Standard GS-FLX amplicon sequencing protocol. The error rate was defined as the number of errors divided by the total number of expected bases. Homopolymeric regions were defined as regions containing repeats of three or more identical bases and the flanking non-identical bases. Data were retrieved from the Amplicon Variant Analysis software (Roche) with an average coverage of 20.000 reads per position. An overall error rate of 0.10% and 0.08% was obtained for pHXB2 and pYK-JRCSF, respectively. In addition, the error rate was elevated in homopolymeric regions: 0.13% *versus *0.07% in non-homopolymeric regions. Of the observed errors, deletions (~40%) and insertions (~33%) were the most frequent error types, followed by substitutions (~26%). These errors were not evenly distributed: homopolymeric regions contained more deletions than non-homopolymeric regions, while the latter contained more insertions and substitutions. The maximum substitution error rate per base position was 0.13%, transitions from A to G were most common, but also T to C, G to C, and C to G were frequently seen. A similarity was noted in frequency errors between duplicates, whereas differences between the two plasmids could be explained by sequence context. It is safe to state that viral variability can be reliably differentiated from procedural/experimental errors in a range of 5- to 10- fold higher. Therefore, mutations were accepted as real variants when present at a frequency above 1% of the total number of reads. In cases when the total number of reads was falling below 5,000, a fixed cut-off of 50 reads is used.

### Phenotyping of the NH2-V4 region using the Virco Tropism Assay (VTA)

Phenotyping was performed as described [12]. Briefly, NH_2_-V4 gp120 amplicons were generated by one-step RT-PCR using primers Env-6210F and Env-R3. NH_2_-V4 gp120 amplicons were purified, and cloned into pHXB2D-ΔNH_2_-V4-eGFP by *in vitro *recombination using the In-Fusion™ Dry-Down PCR Cloning Kit (Clontech-Westburg, Leusden, The Netherlands). Recombination mixes were transformed into MAX Efficiency^® ^Stbl2™ cells (Invitrogen), and recombinant plasmids were purified and transfected into 293T cells using the Amaxa nucleofection technology (Amaxa Biosystems, Cologne, Germany). Transfected cells were cultured for 48 h after which recombinant virus stocks were harvested. 100 μl recombinant virus stock was added to U87-CD4, U87-CD4-CXCR4 and U87-CD4-CCR5 cells. After five days, infection was evaluated by eGFP expression analysis using an argon laser-scanning microscope.

## Competing interests

IVDB, HVM, WM, VVE, ER, KT, KVB, and LJS are employees of Tibotec Virco Virology BVBA. The company is marketing the following HIV diagnostic assays: vircoTYPE and Antivirogram. However, the assays described in this manuscript are only research tools and are not developed for commercial activities of the company. There are no competing interests.

## Authors' contributions

IVDB, HVM, WM, VVE, ER, KT, and KVB participated in the design of the assay, the performance of the experiments and interpretation of the results. KF, DV, KK, SDW, EF, and MM are consortium members who contributed in the design of the study, the collection of samples, and discussion on interpretation of results. LVDK, CV and LJS designed the initial study and were involved in the coordination, and interpretation of the results. All authors read and approved the final manuscript.

## Supplementary Material

Additional file 1 **Overview of the results**. a = VTA: Virco tropism assay; R5 = R5-tropic virus; D/M = dual mixed R5/X4 tropic virus. b = total reads divided by copy input. Click here for file
